# Distribution of residual tumors in esophageal squamous cell carcinoma after neoadjuvant PD-1 blockade combined with chemotherapy

**DOI:** 10.3389/fonc.2023.1067897

**Published:** 2023-02-28

**Authors:** Dongxian Jiang, Qi Song, Han Tang, Peng Shi, Xiaolei Zhang, Yufeng Liu, Haixing Wang, Minying Deng, Jie Huang, Jieakesu Su, Chen Xu, Lijie Tan, Yingyong Hou

**Affiliations:** ^1^ Department of Pathology, Zhongshan Hospital, Fudan University, Shanghai, China; ^2^ Shanghai Institute of Infectious Disease and Biosecurity, Fudan University, Shanghai, China; ^3^ Department of Thoracic Surgery, Zhongshan Hospital, Fudan University, Shanghai, China; ^4^ Center for Evidence-based Medicine, Fudan University, Shanghai, China; ^5^ Pediatric Clinical Research Unit, Department of Research Management, Children’s Hospital of Fudan University, Shanghai, China

**Keywords:** residual tumor, distribution, anti-PD-1 therapy, pathological response, esophageal squamous cell carcinoma

## Abstract

**Aims:**

The distribution of residual esophageal squamous cell carcinoma (ESCC) in the esophageal wall and resected lymph nodes was evaluated after neoadjuvant chemoimmunotherapy (nICT).

**Methods and results:**

Clinical data were collected from 137 ESCC patients who underwent anti-programmed death 1 therapy and esophagectomy. Ninety (65.7%) achieved an major pathological response (MPR) in the esophageal wall, and 27 (19.7%) achieved an MPR in the lymph nodes. Pathologically complete response (pCR, ypT0N0) was observed in 26 patients (19%). Residual tumors located in the mucosa and/or submucosa were found in 94.6% of nonpCR patients. In the minor responders, 97.8% had residual tumor >10% in the mucosa or submucosa. A preferential regression direction toward the lumen was found in 76.4% of prepT2 nonpCR patients, or 60.7% of prepT3-4a nonpCR patients. The correlation between pCR in the esophageal wall and in lymph nodes was not significant (*P*=0.143). Among 19 patients with pCR in resected recurrent laryngeal nerve (RLN) lymph nodes, 31.6% had residual tumor cells in other resected lymph nodes. A significant correlation was found between ypT/ypN downstaging and tumor regression grade (*P*<0.05).

**Conclusions:**

After nICT for ESCC, residual tumors were frequently found in the mucosa or submucosa, with relatively high responsiveness of the invasive front and a significant correlation with downstaging, which may help clinicians make appropriate decisions about postoperative treatment and surveillance. The differences in pCR status in primary tumors, resected lymph nodes, and RLN lymph nodes indicated the importance of assessing regression changes in all resected lymph nodes during clinical practice.

## 1 Introduction

Esophageal cancer (EC) is the seventh most common malignancy and the sixth leading cause of cancer death worldwide (1). EC can be broadly divided into two histologic subtypes: esophageal squamous cell carcinoma (ESCC) and esophageal adenocarcinoma. ESCC is the most frequent histologic subtype in China, where the number of new cases and related deaths reached 246,000 and 188,000, respectively, in 2021 (2). Based on randomized controlled trials (RCTs), neoadjuvant chemoradiotherapy (nCRT) or chemotherapy (nCT) followed by surgery has been regarded as standard treatment for patients with locally advanced ESCC (3, 4). Despite efforts over the past decade to improve the survival of advanced-stage ESCC, overall survival remains dismal. Recently, immunotherapy against programmed death 1 (PD-1) or programmed death ligand 1 (PD-L1) represents a relatively innovative treatment for malignant tumors, showing particular efficacy and a low toxicity profile in advanced or metastatic ESCC (5). The treatment response and safety of PD-1 inhibitors combined with chemotherapy have generated interest in extending their use into neoadjuvant therapy (6, 7).

Histopathological response was used as a surrogate endpoint for the clinical efficacy of neoadjuvant therapy, including neoadjuvant immunotherapy (8, 9). Although there are well-established differences in molecular mechanisms and microscopic appearance between immunotherapy and chemotherapy, tumor regression grading (TRG) systems are widely used in clinical practice (10). Numerous studies have shown that no residual tumor (pathological complete response, pCR) or no more than 10% residual viable tumor indicates a major pathological response (MPR), which is ideal for predicting the long-term survival in ESCC patients (11). In ESCC after nCRT or nCT, several published studies have shown some findings and conclusions. More residual tumors were involved in the mucosa and submucosa, and the overall regression pattern was most frequently a mixed pattern of both concentric regression and regression toward the lumen (12). The MPR of the primary tumor may not be the same as that of lymph node metastases (13). The recurrent laryngeal nerve (RLN) lymph nodes are a crucial indicator during esophagectomy (14). The initial clinical staging of both tumor invasive depth and lymph node metastasis was downstaged (15). Whether the aforementioned observations in nCRT or nCT may analogously be extended to locally advanced-stage ESCC treated with PD-1 inhibitors combined with chemotherapy has not been reported.

Therefore, this retrospective study was conducted to 1) describe the exact location and distribution of residual tumor in the esophageal wall and resected lymph nodes after neoadjuvant chemoimmunotherapy (nICT); 2) describe the tumor regression pattern of ESCC as induced by nICT; 3) describe the pathological response in resected lymph nodes, including RLN lymph nodes; and 4) evaluate ypT or ypN downstage and their association with pathological response.

## 2 Materials and methods

### 2.1 Patients

Patients with locally advanced esophageal cancer who had undergone a combination therapy of PD-1 inhibitors and chemotherapy (nICT) followed by surgery at Zhongshan Hospital, Fudan University between March 2020 and October 2021 were enrolled. The eligibility criteria were as follows: 1) patients pathologically diagnosed with ESCC using biopsy specimens prior to receiving nICT; 2) patients who received a scheduled complete course of nICT; and 3) patients who underwent total or subtotal thoracic esophagectomy with regional lymph node dissection after nICT. The exclusion criteria were as follows: 1) patients with unresectable tumors (T4b) and/or distant metastasis (M1) and 2) patients with tumors clinically limited to the mucosa or the submucosa (T1). Pretreatment staging was performed using computed tomography (CT) of the neck, chest, and abdomen, endoscopic ultrasound (EUS), and positron emission tomography (PET). All patients were staged according to the 8th edition of the American Joint Committee on Cancer staging manual. Consequently, 137 ESCC patients were enrolled in the study. This study protocol was approved by the institutional review board of Zhongshan Hospital, Fudan University (B2022-632).

### 2.2 Neoadjuvant PD-1 inhibitors and chemotherapy

The regimen of nICT consisted of anti-PD-1 therapy with pembrolizumab (200mg), camrelizumab (200mg), toripalimab (240mg), or sintilimab (200mg) on Day 1. The chemotherapy regimen consisted of cisplatin (75mg/m^2^, d1-2) and albumin bound paclitaxel (125mg/m^2^, d1, 8). Two to four courses of nICT were used, separated by a 3-week interval. All patients were scheduled to receive endoscopic examination and CT after each course of the above therapy to evaluate the therapeutic effect.

Surgical resection was performed 3-5 weeks after the completion of anti-PD-1 and chemotherapy. Our standard procedures consisted of the McKeown procedure for upper, middle, or lower esophageal tumors and the Ivor-Lewis procedure for middle or lower tumors, with at least 2-field lymphadenectomy. For tumors that were not located in the upper third of the esophagus, cervical lymphadenectomy was performed according to pretreatment radiological diagnosis and intraoperative pathological diagnosis of metastasis in the RLN lymph nodes.

### 2.3 Pathological examination

All resection specimens were initially processed using a pathologically standardized protocol. In particular, all tumors were sampled and embedded. When the macroscopic appearance was not obvious, subtle lesions, such as an ulcer or an irregular area, together with surrounding areas were sampled to adequately evaluate the location and distribution of the residual tumor. In cases where only patchy residual tumors were present and their distribution was not contiguous, large (mega) blocks were used to show the complete cross-section of the esophageal wall.

All HE-stained sections were scanned at ×40 magnification using a NanoZoomer S360 Digital Slide Scanner C13220-01(HAMAMATSU PHOTONICS, Hamamatsu city, Japan) and systematically reviewed by an experienced gastrointestinal pathologist with NanoZoomer digital slide viewing software. The histomorphologic assessment included evaluation of all esophageal walls and resected lymph nodes.

The original tumor area was measured based on a series of regression changes (**Supplementary Figure 1**), such as giant cell reaction around ghost cells and keratin pearls, foamy histiocytes, cholesterol deposits, foreign body reaction, calcifications, fibrosis, granulation, inflammatory, and vascular change, compared with surrounding normal tissues (9, 11). Given these measurements, the original depth of the primary tumor and the plausible number of metastatic lymph nodes were recorded as prepT and prepN (16).

### 2.4 Pathological evaluation

**Figure 1:**
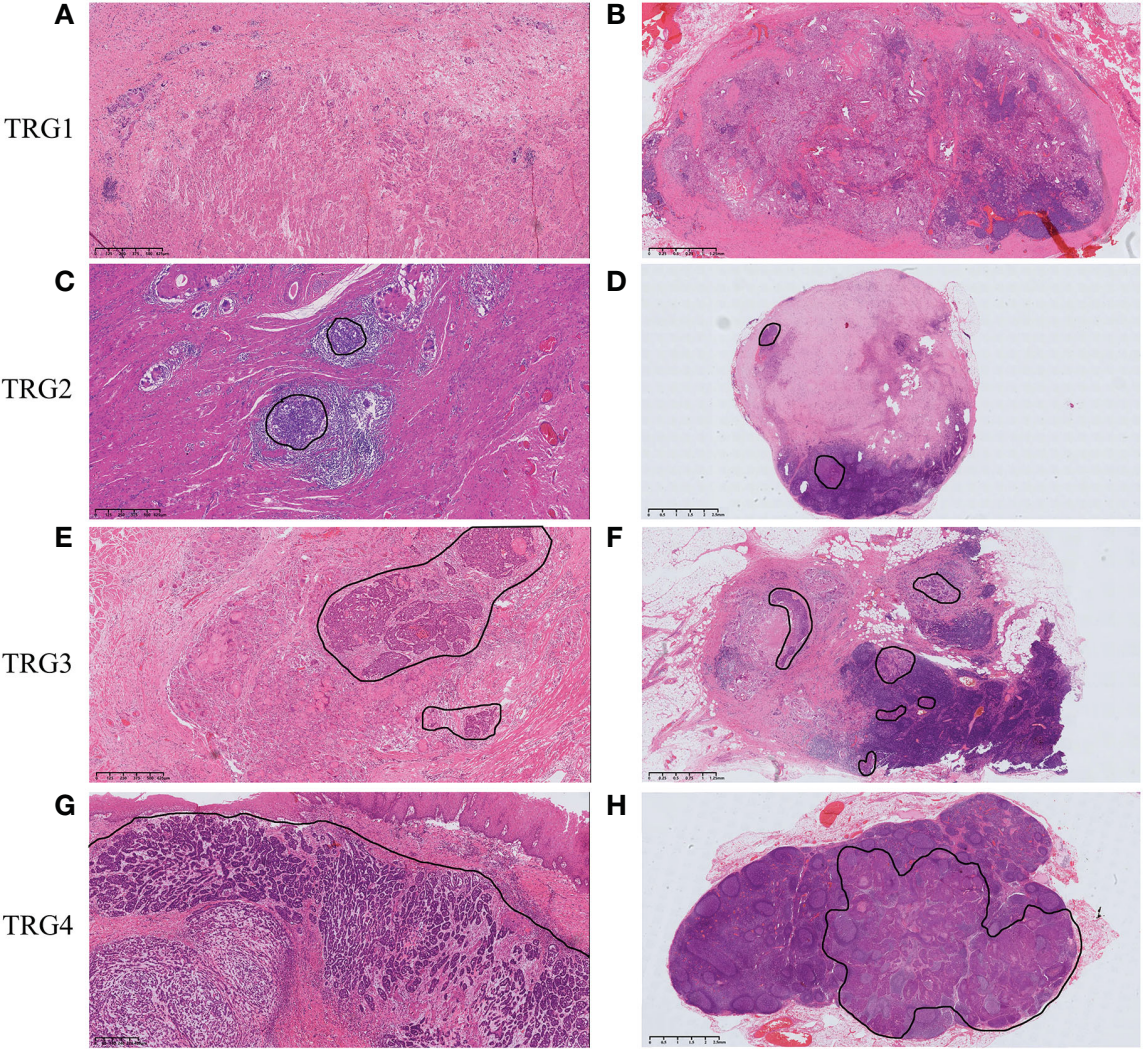
Tumor regression grading according to Chirieac et al. in ESCC. **(A)** TRG1 in esophageal wall, no residual carcinoma in esophageal wall. **(B)** TRG1 in lymph nodes, no residual carcinoma in resected lymph nodes. **(C)** TRG2 in esophageal wall, 1% to 10% residual carcinoma in esophageal wall. **(D)** TRG2 in lymph nodes, 1% to 10% residual carcinoma in resected lymph nodes. **(E)** TRG3 in esophageal wall, 11% to 50% residual carcinoma in esophageal wall. **(F)** TRG3 in lymph nodes, 11% to 50% residual carcinoma in resected lymph nodes. **(G)** TRG4 in esophageal wall, greater than 50% residual carcinoma in esophageal wall. **(H)** TRG4 in lymph nodes, greater than 50% residual carcinoma in resected lymph nodes.

TRG was assessed using the modified Mandard scoring system as reported by Chirieac et al (17). TRG was divided into 4 categories: 1) TRG1, no residual carcinoma (pCR); 2) TRG2, 1% to 10% residual carcinoma; 3) TRG3, 11% to 50% residual carcinoma; and 4) TRG4, greater than 50% residual carcinoma. TRG grades (1, 2, 3, and 4) in the metastatic lymph nodes were pathologically determined according to the same criteria (**Figure 1**).

**Figure 2:**
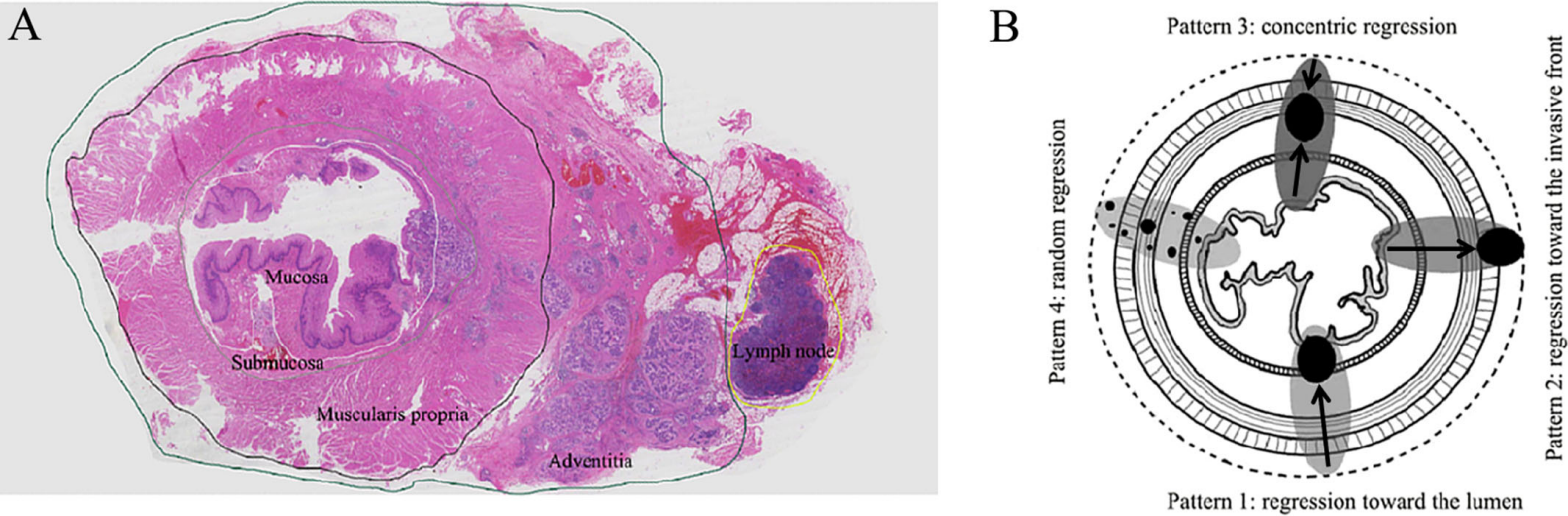
**(A)** The large (mega) blocks showing 4 esophageal wall layers (mucosa, submucosa, muscularis propria, adventitia) and surrounding lymph nodes. **(B)** The four regression patterns in esophageal wall: Pattern 1, regression toward the lumen; Pattern 2, regression toward the invasive front; Pattern 3, concentric regression; and Pattern 4, random regression.

In 4 esophageal wall layers (mucosa, submucosa, muscularis propria, adventitia) and all resected lymph nodes, TRG was evaluated and scored individually in all slides containing regression changes and/or residual tumor (**Figure 2A**). An average TRG of the esophageal wall was calculated for each individual by averaging the TRG score of each layer in all slides. An average TRG of lymph nodes was also calculated by averaging the TRG score of all plausible positive metastatic lymph nodes.

The relative distribution of residual tumor cells within the 4 esophageal wall layers and lymph nodes was assessed using the methods reported by Shapiro et al (12). Comparing the percentage distribution of residual tumor in 2 wall layers with the other 2 layers, a higher percentage indicated less regression. The regression directions were from layers with more regression to layers with less regression and divided into 4 patterns (**Figure 2B**): 1) regression toward the lumen, less regression in the mucosa and/or the submucosa; 2) regression toward the invasive front, less regression in the muscularis propria and/or adventitia; 3) concentric regression, less regression in the submucosa and/or muscularis propria; and 4) random regression, comparable extent of regression in all layers.

### 2.5 Statistical analysis

All statistical analyses were conducted using SPSS software, version 21.0 (SPSS Inc., Chicago, IL, USA). Categorical variables are presented by frequency n (%), and continuous variables with nonnormal distribution are presented as median values with ranges. Intergroup comparisons were performed using the chi-square test or Fisher’s exact test for categorial variables. To compare the distributions of pCR percentage percentages, the McNemar test was used for related binary results. All *P* values were reported as 2-sided, with a significance level of 0.05.

## 3 Results

### 3.1 Patient characteristics7

**Table 1**summarizes the baseline characteristics of 137 ESCC patients with nICT. The median age was 63 years, and the majority of patients were male (84.7%). Most patients were in the lower third of the esophagus (n=63, 46%) and underwent the McKeown surgical approach (n=104, 75.9%) or Ivor-Lewis procedure (n=33, 24.1%) in a median of 35 days between neoadjuvant therapy and surgery. The original tumor area was characterized and identified as follows: 1) dense immune infiltrates, such as tertiary lymphoid structure (TLS) and dense tumor infiltrating lymphocyte (TIL) infiltrates; 2) cell death, such as cholesterol clefts and interstitial foamy macrophages; and 3) tissue repair, such as neovascularization and proliferative fibrosis (**Supplementary Figure 1**). According to the regression bed, 55.5% of patients had stage prepT2 disease, 43.8% had stage prepT3 disease, and 0.7% had stage prepT4a disease. The prepN1-3 were found in 69 (50.4%) patients. The median number of identified and evaluated lymph nodes was 22 (range, 5-57). A total of 31 patients (22.6%) had no vital tumor cells in the esophageal wall after nICT (ypT0), and 90 patients (65.7%) had no pathological lymph nodes (ypN0). The MPR was 65.7% in the esophageal wall and 19.7% in the lymph nodes. Overall pCR (ypT0N0) was observed in 26 patients (19%).

**Table 1:** Patient characteristics at baseline.

Characteristic	N	%
Median age (range), yr	63 (45-77)	
Sex		
Female	21	15.3
Male	116	84.7
Location		
Upper	31	22.6
Middle	43	31.4
Lower	63	46
prepT stage		
prepT2	76	55.5
prepT3	60	43.8
prepT4a	1	0.7
prepN stage		
prepN0	68	49.6
prepN1	36	26.3
prepN2	26	19
prepN3	7	5.1
Median number of lymph nodes resected (range)	22 (5-57)	
ypT stage		
ypT0	31	22.6
ypT1	40	29.2
ypT2	30	21.9
ypT3	35	25.5
ypT4a	1	0.7
ypN stage		
ypN0	90	65.7
ypN1	29	21.2
ypN2	15	10.9
ypN3	3	2.2
TRG-eophageal wall		
TRG1	31	22.6
TRG2	59	43.1
TRG3	36	26.3
TRG4	11	8
TRG-lymph node		
TRG1	22	16.1
TRG2	5	3.6
TRG3	15	10.9
TRG4	27	19.7
pCR		
No	111	81
Yes	26	19

### 3.2 Location and distribution of residual tumors in nonpCR patients

In addition to 26 pCR patients, 111/137 (81.0%) patients had residual tumor cells remaining after nICT (nonpCR group) (**Supplementary Figure 2**), which were used for further analysis of the localized residual disease. Among the 111 cases, 55 patients (49.5%) had regression changes and/or residual tumor reaching into the muscularis propria (prepT2), and 56 (50.5%) showed regression changes and/or residual tumor reaching into the adventitia (prepT3-4a). Among the 111 cases, a total of 59 (53.2%) patients had regression changes and/or residual tumor in lymph nodes (prepN1-3).

**Figure 3:**
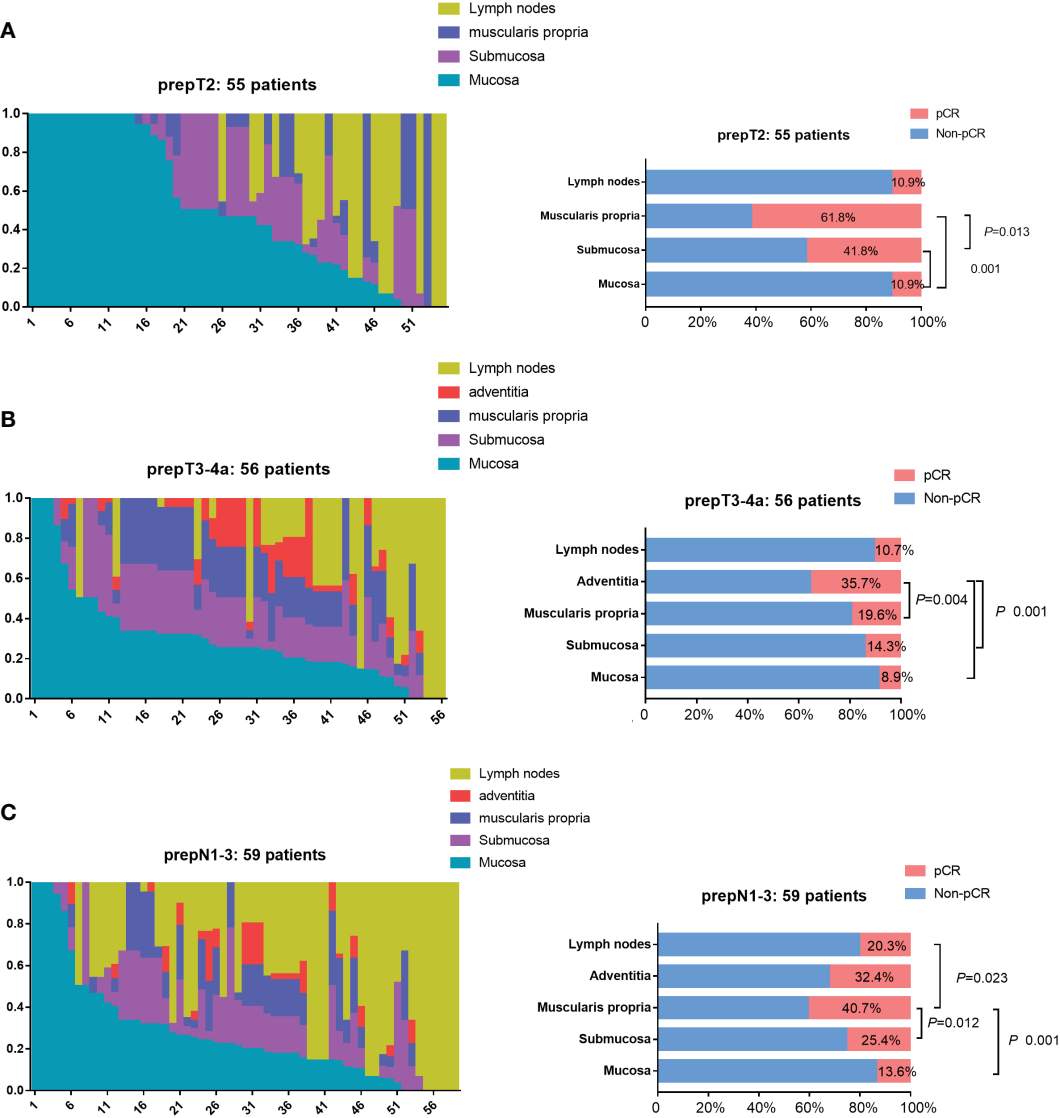
Residual tumor location in nonpCR patients. **(A)** The distribution of residual tumor cells within 3 esophageal wall layers (mucosa, submucosa, muscularis propria) and lymph nodes in 55 prepT2 nonpCR patients. Residual tumors in the mucosa and/or submucosa were found in 52 (94.6%) of 55 patients. The mucosa had residual tumor cells significantly more frequently than the submucosa and muscularis propria (*P*<0.001). **(B)** The distribution of residual tumor cells within 4 esophageal wall layers (mucosa, submucosa, muscularis propria, adventitia) and lymph nodes in 56 prepT3-4a nonpCR patients. Residual tumors in the mucosa and/or submucosa were found in 53 (94.6%) of 56 patients. The mucosa, submucosa, and muscularis propria had residual tumor cells significantly more frequentlythan the adventitia (*P*<0.005). **(C)** The distribution of residual tumor cells within 4 esophageal wall layers (mucosa, submucosa, muscularis propria, adventitia) and lymph nodes in 59 N1-N3 nonpCR patients. Residual tumors in the mucosa and/or submucosa were found in 54 (91.5%) of 59 patients. The mucosa, submucosa, and lymph nodes had residual tumor cells significantly more frequently than the muscularis propria (P<0.05).

In the prepT2 nonpCR group, 89.1% (49/55) of patients had residual tumor cells in the mucosa, 58.2% (32/55) in the submucosa, 38.2% (21/55) in the muscularis propria, and 89.1% (6/25) in the lymph nodes. **Figure 3A** provides the relative distribution of residual tumor within the esophageal wall and lymph nodes in 55 patients. The mucosa had residual tumor cells significantly more frequently than the submucosa and muscularis propria (*P*<0.001). Residual tumors in the mucosa and/or submucosa were found in 52 (94.6%) of 55 patients. For the remaining 3 patients, 1 (1.8%) had residual tumor involving the muscularis propria, and 2 (3.6%) patients had residual tumor only in lymph nodes.

In the prepT3-4a nonpCR group, 91.1% (51/56) of patients had residual tumor cells in the mucosa, 85.7% (48/56) in the submucosa, 80.4% (45/56) in the muscularis propria, 64.3% (36/56) in the adventitia, and 89.3% (28/34) in the lymph nodes. **Figure 3B** provides the relative distribution of residual tumor within the esophageal wall and lymph nodes in 56 patients. The mucosa, submucosa, and muscularis propria had residual tumor cells significantly more frequently than the adventitia (*P*<0.005). Residual tumors in the mucosa and/or submucosa were found in 53 (94.6%) of 56 patients. The remaining 3 patients had residual tumors only in the lymph nodes.

In the prepN1-3 nonpCR group, 86.4% (51/59) of patients had residual tumor cells in the mucosa, 74.6% (44/59) in the submucosa, 59.3% (35/59) in the muscularis propria, 67.6% (23/34) in the adventitia, and 79.7% (47/59) in the lymph nodes. **Figure 3C** provides the relative distribution of residual tumor within the esophageal wall and lymph nodes in 59 patients. The mucosa, submucosa, and lymph nodes had residual tumor cells significantly more frequently than the muscularis propria (*P*<0.05). Residual tumors in the mucosa and/or submucosa were found in 54 (91.5%) of 59 patients. The remaining 5 patients had residual tumors only in the lymph nodes.

### 3.3 Minor responders in the mucosa and submucosa

**Figure 4:**
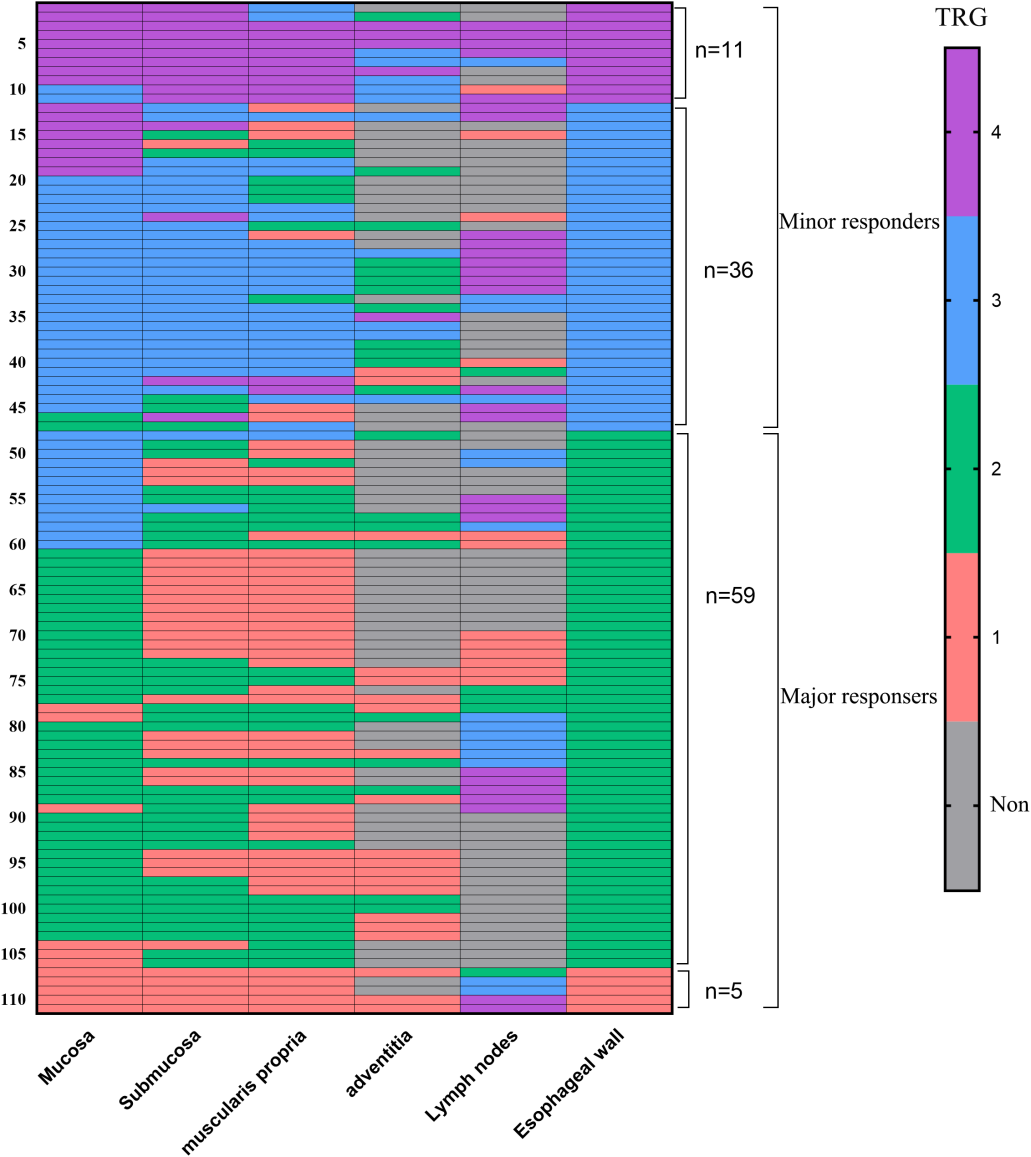
The tumor regression grading (TRG) within 4 esophageal wall layers (mucosa, submucosa, muscularis propria, adventitia) and lymph nodes in minor responders and major responsers. 97.8% (45 plus 1) of the minor responders had residual tumors>10% (TRG3 and TRG4) in the mucosa or submucosa, and 22.0% (13 plus 0) of the major responses had residual tumors>10% (TRG3 and TRG4) in the mucosa or submucosa.

In the nonpCR group, 36 (32.4%) patients had an esophageal wall TRG3 response, and 11 (9.9%) patients had an esophageal wall TRG4 response, which were regarded as minor responders. Of these 47 esophageal wall minor responders, 45 (95.7%) patients also showed a minor response in the mucosa, 41 (87.3%) in the submucosa, 34 (72.3%) in the muscularis propria, 15 (15/29, 51.7%) in the adventitia, and 21 (21/26, 80.8%) in the lymph nodes. **Figure 4** shows that 45 of 47 esophageal wall minor responders showed a minor response in the mucosa, 1 showed a minor response in the submucosa and lymph nodes, and 1 showed a minor response in the muscularis propria. Therefore, 97.8% (45 plus 1) of the minor responders had residual tumors>10% in the mucosa or submucosa.

In the nonpCR group, 59 (53.2%) patients had an esophageal wall TRG2 response. Among the 59 major responses, 13 (22.0%) patients had TRG3 or TRG4 in the mucosa, 2 (3.4%) in the submucosa, 1 (1.7%) in the muscularis propria, 0 (0/24, 0%) in the surrounding stroma, and 17 (17/28, 60.7%) in the lymph nodes (**Figure 4**). **Figure 4** shows that 22.0% (13/59) of the major responses had residual tumors>10% in the mucosa or submucosa.

### 3.4 Tumor regression pattern in the esophageal wall

**Figure 5:**
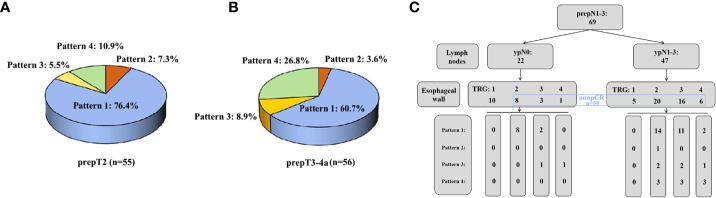
**(A)** The percentage of four regression patterns in 55 prepT2 nonpCR patients. **(B)** The percentage of four regression patterns in 56 prepT3-4a nonpCR patients. **(C)** The distribution of four regression patterns in 69 prepN1-3 patients.

In the prepT2 nonpCR group, the mucosa did show a significantly lower percentage of TRG1 (pCR, 10.9%) than the submucosa (41.8%; *P*<0.001) and muscularis propria (61.8%; *P*<0.001). The muscularis propria also differed significantly from the submucosa (41.8%; *P*=0.013). The overall regression pattern was analyzed: regression toward the lumen (76.4%) was significantly more common than regression toward the invasive front (7.3%), concentric regression (5.5%), and random regression (10.9%) (*P*<0.001) (**Figure 5A**). The random regression pattern (10.9%) was much less common than a nonrandom regression pattern (89.1%) (*P*<0.001).

In the prepT3-4a nonpCR group, the mucosa did show a significantly lower percentage of TRG1 (pCR, 8.9%) than the adventitia (35.7%, *P*<0.001) but not the submucosa (14.3%, *P*=0.257) or muscularis propria (19.6%, *P*=0.058). The adventitia also differed significantly from the submucosa (*P*=0.001) but not the muscularis propria (*P*=0.083). Regression toward the lumen (60.7%) was significantly more common than regression toward the invasive front (3.6%, *P*<0.001), concentric regression (8.9%, *P*<0.001), and random regression (26.8%, *P*=0.010) (**Figure 5B**). The random regression pattern (26.8%) was much less common than a nonrandom regression pattern (73.2%) (*P*=0.001).

### 3.5 Tumor regression in prepN1-3 patients

In the prepN1-3 group, 22 (31.9%) of 69 patients had a pCR (TRG1) in all resected lymph nodes, whereas 47 (68.1%) patients had residual tumor cells in one or more of the resected lymph nodes. The percentage of pCR in regional lymph nodes was higher than the percentage of overall pCR (14.5%, 10/69, *P*<0.001). Ten of 22 (45.5%) patients had an esophageal wall pCR, 8 of 22 patients (36.4%) had an esophageal wall TRG2, 3 of 22 patients (13.6%) had an esophageal wall TRG3, and 1 of 22 patients (4.5%) had an esophageal wall TRG4. Among 47 patients with ypN1-3, 5 (10.6%) patients had an esophageal wall pCR, 20 patients (42.6%) had an esophageal wall TRG2, 16 patients (34.0%) had an esophageal wall TRG3, and 6 patients (12.8%) had an esophageal wall TRG4. Regression toward the lumen was also more common in patients with 22 pCR lymph nodes (83.3%) or 47 nonpCR lymph nodes (64.3%) (**Figure 5C**). The correlation between pCR in the esophageal wall and pCR in lymph nodes was not significant (*P*=0.143).

Among 69 prepN1-3 patients, 45 (65.2%) had regression changes and/or residual tumor in RLN lymph nodes. Nineteen (42.2%) of 45 patients had TRG1 in all resected RLN lymph nodes, whereas 26 (57.8%) patients had residual tumor cells in one or more of the resected lymph nodes. Among 19 patients with TRG1 in all resected RLN lymph nodes, 6 (31.6%) patients had residual tumor cells in one or more of the other resected lymph nodes (**Table 2**). The correlation between the pCR in RLN lymph nodes and pCR in all lymph nodes was significant (*P*<0.001).

**Table 2:** Tumor regression grading (TRG) in all resected lymph nodes and recurrent laryngeal nerve lymph nodes.

			All resected lymph nodes			
	TRG	n	1	2	3	4
Recurrent laryngeal nerve lymph nodes	1	19	13	2	2	2
	2-4	26	0	3	8	15

### 3.6 ypStage and TRG

The numbers of patients with prepT2, prepT3, and prepT4a were 76, 60, and 1, respectively. **Supplementary Figure 3A** provides the distribution of TRG in the esophageal wall with or without ypT downstage. 1) Of the 76 prepT2 patients, 23 were ypT0, 32 were ypT1, and 21 were ypT2, which showed that 72.4% of tumors were downstaged. In patients in the downstaged group, 41.8% (23/55), 47.3% (26/55), and 10.9% (6/55) of patients had esophageal wall TRG1, TRG2, and TRG3, respectively. In patients in the nondownstaged group, 42.8% (9/21), 52.4% (11/21), and 4.8% (1/21) of patients had esophageal wall TRG2, TRG3, and TRG4, respectively. A significant difference was found between the two groups (*P*<0.001). 2) Of the 60 prepT3 patients, 8 had ypT0, 8 had ypT1, 9 had ypT2, and 35 had ypT2, which showed that 41.7% of tumors were downstaged. In patients in the downstaged group, 32.0% (8/25), 60.0% (15/25), and 8.0% (2/25) of patients had esophageal wall TRG1, TRG2, and TRG3, respectively. In patients in the nondownstaged group, 25.7% (9/35), 45.7% (16/35), and 28.6% (10/35) of patients had esophageal wall TRG2, TRG3, and TRG4, respectively. A significant difference was also found between the two groups (*P*<0.001). 3) One prepT4a was also ypT4a, with esophageal wall TRG3.

The numbers of patients with prepN1, prepN2, and prepN3 were 36, 26, and 7, respectively. **Supplementary Figure 3B** provides the distribution of TRG in lymph nodes with or without N downstage. 1) Of the 36 prepN1, 19 were ypN0, and 17 were ypN1, which showed that 52.8% of tumors were downstaged. In patients in the downstaged group, all (19/19) patients had lymph node TRG1. In patients in the nondownstaged group, 5.8% (1/17), 47.1% (8/17), and 47.1% (8/17) of patients had lymph nodes TRG2, TRG3, and TRG4, respectively. 2) Of the 26 prepN2 cases, 3 were ypN0, 11 were ypN1, and 12 were ypN2, which showed that 53.8% of the tumors were downstaged. In the downstaged group, 21.4% (3/14), 14.3% (2/14), 35.7% (5/14), and 28.6% (4/14) of patients had lymph nodes TRG1, TRG2, TRG3, and TRG4, respectively. In the nondownstaged group, 8.3% (1/12) and 91.7% (11/12) of patients had lymph nodes TRG2 and TRG4, respectively. 3) Of the 7 prepN3 patients, 1 was ypN1, 3 were ypN2, and 3 were ypN3, which showed that 57.1% of tumors were downstaged. In the downstaged group, 25.0% (1/4), 25.0% (1/4), and 50.0% (2/4) of patients had lymph nodes TRG2, TRG3, and TRG4, respectively. In the nondownstaged group, 33.3% (1/3) and 66.7% (2/3) of patients had lymph nodes TRG3 and TRG4, respectively.

## 4 Discussion

This study includes one of the largest cohorts of ESCC patients treated with neoadjuvant nICT at a single institution. The MPR in the esophageal wall was 43.1%, which is in accordance with published reports on this subject, in which values range from 41.7% to 50.0% (18, 19). The overall pCR rate was 19%, with 22.6% pCR in the esophageal wall and 31.9% pCR in the resected lymph nodes. Our overall pCR rate was consistent with two recent reports from He et al. (18.8%, 3/16, from Chengdu) (20) and Hong et al. (18.8, 6/32, from Fuzhou) (21). However, previous published reports by Liu et al. and Yang et al.from Guangzhou showed a pCR rate of 35.3% (18/51) (22) and 25.0% (5/23) (19). The difference in pCR rates might be attributed to the sample size and the difference in enrolled population. Some prospective large-scale studies to determine the benefit and safety of nICT are needed in the future.

### 4.1 More residual tumor in mucosa or submucosa

The location of the bulk of residual tumor in mucosa, submucosa, muscularis propria and adventitia was analyzed. A study from Shapiro et al., which examined the distribution of residual cancer cells within the esophageal wall after nCRT, found that 89% (63/71) of EC patients had residual tumors present in the mucosa, submucosa, or both (12). In our previous study, 83.3% (115/138) of ESCC patients had residual tumors in these two superficial layers after nCRT (16). These results indicated the preferential persistence of malignant cells in the mucosa/submucosa of EC after nCRT. In terms of the differences in molecular mechanism and microscopic appearance between immunotherapy and chemotherapy, we wondered whether there were some differences in the location and distribution of residual tumors. In this nICT study, whether in the prepT2 nonpCR group or in the prepT3-4a nonpCR group, the percentage of residual tumors in the mucosa and/or submucosa was 94.6%, which was slightly higher than those with nCRT.

To detect residual tumors during neoadjuvant therapy, endoscopy and CT are the current standards, and additional examinations, such as PET, EUS, or biopsy, may be added (23). In our study, 94.6% of residual tumors were in the mucosa and/or submucosa. Moreover, 97.8% of the minor responders had residual tumors>10% in the mucosa or submucosa. This indicated that there was a higher chance of detecting residual tumors in the mucosa or submucosa. With improvements in the efficacy of detecting residual tumors by endoscopy biopsies or fine-needle aspirations, it might be possible to detect residual disease in mucosa or submucosa for restage after nICT and subsequent surveillance (24). Some patients who want to have organs preserved might have the chance to consider a wait-and-see approach (25).

### 4.2 The directions of regression toward the lumen

By comparing the residual tumors in 2 esophageal wall layers with those in the other 2 wall layers, the directions of regression were categorized into four patterns in nCRT: 1) regression toward the lumen; 2) regression toward the invasive front; 3) concentric regression; and 4) random regression. In Shapiro et al.’s study, the regression pattern was mainly a mixed pattern of concentric regression and regression toward the lumen (12). In our previous study, random regression (49%) was significantly more common than the other 3 types (19%, 15%, and 17%) (16). Here, we compared the 4 regression patterns in patients with ESCC after nICT. In the prepT2 patients and prepT3-4a patients, regression toward the lumen (76.4%/60.7%) was significantly more common than the other 3 patterns (7.3%/3.6%, 5.5%/8.9%, and 10.9%/26.8%). Our results indicated the relatively high responsiveness of the invasive front (muscularis propria and/or adventitia) in ESCC after nICT.

This difference in response to nICT in different layers of the esophageal wall could possibly be explained by cancer cell-stroma interactions. Pathologic features of the response to neoadjuvant anti-PD-1 therapy in non-small-cell lung carcinoma (NSCLC) demonstrated that regression beds surrounded residual tumors and abutted normal background lung tissue (9). In our study, tumors in the invasive front were characterized by dense immune infiltrates and cell death, as reported in other tumors (26). The tumor microenvironment (TME) plays an important role in determining cancer cell sensitivity to PD-1 inhibitors (27). Thus, a better understanding of the TME, such as tumor PD-L1 expression, tertiary lymphoid structure (TLS), tumor-infiltrating lymphocytes (TIL), tumor-associated macrophages (TAM), and myeloid-derived suppressor cells (MDSC), is increasingly important (28–30). In the near future, more mechanistic studies on the interaction between tumor cells and the TME should be conducted to select ESCC patients with the highest chance of benefiting from immunotherapy.

### 4.3 Pathological response in resected lymph nodes

Some regression grading systems included therapy-induced effects on the primary tumor; however, they did not separately assess the responses on resected lymph nodes (11). A study from the Netherlands Cancer Registry that included 645 ESCC patients who underwent nCRT found that high lymph node dissection was associated with improved OS (31). Some prospective observation trials demonstrated that ypN status was a significant prognostic parameter for patients with R0 resection following nCRT (13, 32, 33). In negative nodes, some studies found that the presence of tumor regression changes seems to impact the prognosis and recommended that these should also be included in pathology reports (34). Therefore, although the assessment of the primary tumor was prognostically meaningful, the assessment of the lymph nodes was equally important and significant. In our study, 53.2% (59/111) of patients had regression changes and/or residual tumor in resected lymph nodes (prepN1-3). We found some differences in terms of pCR between primary tumors and lymph nodes. Among 47 patients with residual tumor in lymph nodes (ypN1-3), 10.6% patients had an esophageal wall pCR. Among 22 patients with pCR in lymph nodes (prepN1-3 and ypN0), only 45.5% of patients had an esophageal wall pCR. The pCR rate in lymph nodes (31.9%) was higher than the pCR rate in the esophageal wall (22.6%). At present, there have been few studies evaluating the correlation between lymph node response and the esophageal wall in ESCC after nICT.

RLN lymph nodes, with a reported metastasis rate of 18-63%, are the crucial indicator during esophagectomy, which may have decided the necessity of cervical lymphadenectomy in some previous studies (35). The need to dissect RLN lymph nodes in patients who have undergone neoadjuvant therapy is even more controversial, given that neoadjuvant therapy may clear the metastatic focus and induced mediastinal fibrosis may increase the risk of recurrent laryngeal nerve palsy (14, 36). However, no data are currently available on the treatment response of recurrent laryngeal nerve lymph nodes after nICT. In light of these knowledge gaps, we observed regression changes in RLN lymph nodes after nICT. Among 19 patients with pCR in resected RLN lymph nodes (42.2% of patients with regression changes and/or residual tumor in RLN lymph nodes), 31.6% patients had residual tumors in one or more of other resected lymph nodes. As presented in the current analysis, residual tumors could also be found in lymph nodes of other sites in prepN1-3 patients without visible tumors in the RLN lymph nodes. Therefore, radical lymphadenectomy might also be necessary in cases with pCR of resected RLN lymph nodes at intraoperative frozen diagnosis.

### 4.4 Downstaging and pathological response

Disease downstaging seems to be the strongest prognostic factor in neoadjuvantly treated patients. In the 8^th^ edition AJCC TNM stage of ESCC, neoadjuvant pathologic stage groups are recommended, which highlights the importance of the postneoadjuvant stage (37). The pathological response scoring was thought to be less important. Patients with a good response to nCRT or nCT might have high ypT stage and ypN stage, which may be responsible for the lower relevance of survival in multivariate analysis (33). In our study, 72.4% of prepT2 ESCC, 41.7% of prepT3, 52.8% of prepN1, 53.8% of prepN2, and 57.1% of prepN3 were downstaged after nICT. We also found that in the downstaged group, the percentage of pathological responders was higher than that in the nondownstaged group, with 89.1% vs. 42.8 in prepT2, 92% vs. 25.7% in prepT3, 100% vs. 5.8% in prepN1, 35.7% vs. 8.3% in prepN2, and 25.0% vs. 0 in prepN3. The correlation between T downstaging and pathological response might be attributed to the directions of regression toward the lumen in most of our patients. The regression response may complement ypStage and help clinicians make appropriate decisions about postoperative treatment and surveillance strategies for ESCC patients who undergo nICT followed by surgery.

In conclusion, the current study identified that there was a higher percentage of residual tumors in the mucosa or submucosa in ESCC patients treated with nICT, which offered the opportunity for detecting residual disease in the 2 layers during restaging and subsequent surveillance. The direction of regression toward the lumen was most frequent, which indicated the relatively high responsiveness of the invasive front and may lead to the better correlation between ypStage and pathological response. Some differences in pCR status between primary tumors and resected lymph nodes existed, and they were also found between recurrent laryngeal lymph nodes and lymph nodes of other sites, which indicated that the assessment of all resected lymph nodes is necessary and important. Taken together, our results demonstrated the distribution of residual tumors in ESCC after nICT, which may assist clinicians in making better treatment and follow-up strategies after nICT.

## Data availability statement

The original contributions presented in the study are included in the article/**Supplementary Material**. Further inquiries can be directed to the corresponding authors.

## Ethics statement

The studies involving human participants were reviewed and approved by institutional review board of Zhongshan Hospital, Fudan University (B2022-632). The patients/participants provided their written informed consent to participate in this study.

## Author contributions

YH and LT performed study concept and design; DJ, QS, and HT performed development of methodology and writing; YH and LT review and revision of the paper; DJ, QS, HT, PS, XZ, YL, HW, MD, and JH provided acquisition, analysis and interpretation of data, and statistical analysis; JS and CX provided technical and material support. All authors contributed to the article and approved the submitted version.
