# Supplementary material for: Distribution of residual tumors in esophageal squamous cell carcinoma after neoadjuvant PD-1 blockade combined with chemotherapy
Source: Front Oncol. 2023 Feb 28;13:1067897. doi: 10.3389/fonc.2023.1067897 (PMC10012861; doi:10.3389/fonc.2023.1067897)
Supplement: Supplementary file 1 [file DataSheet_1.zip › Supplementary Figures/Supplementary Figures.pdf]

Supplementary Material

1.1 Supplementary Figures

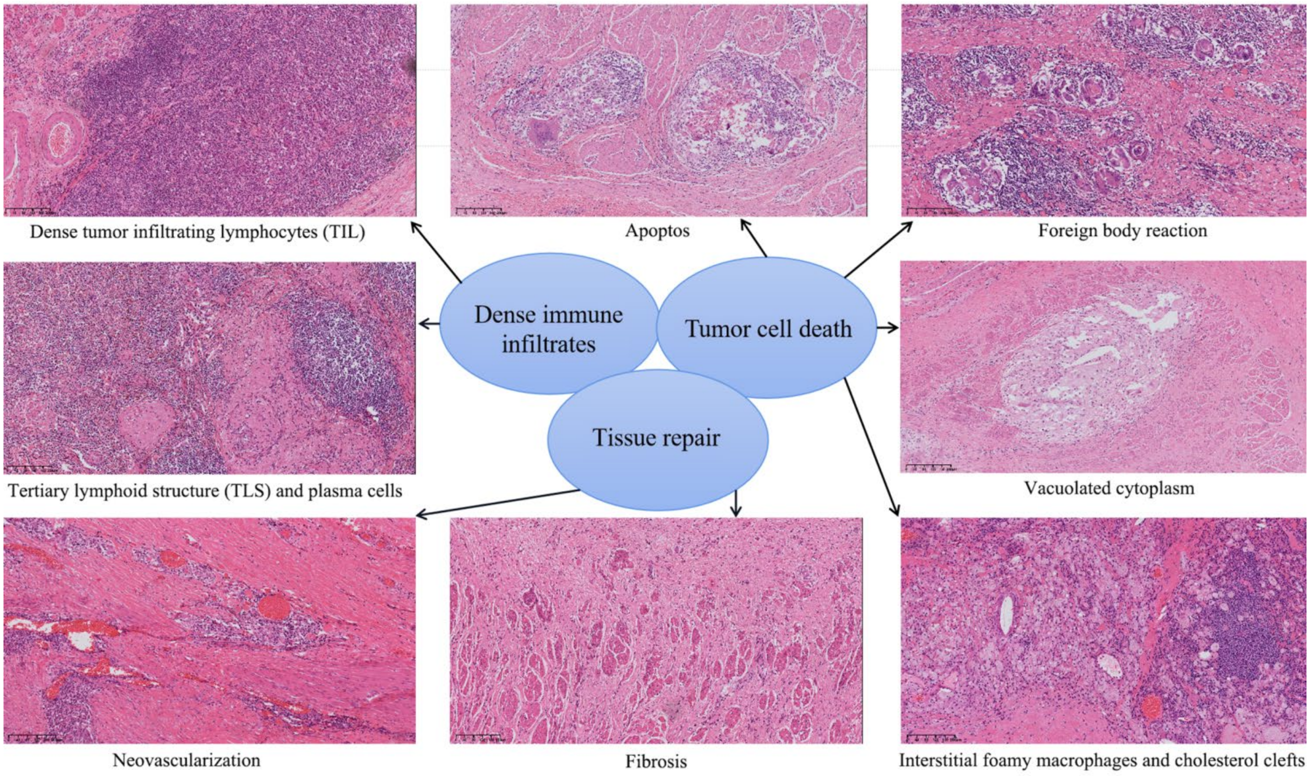

Supplementary Figure 1. Histologic features of pathological response to neoadjuvant chemoimmunotherapy (nICT) in ESCC, characterized by dense immune infiltrates, tumor cell death, and tissue repair.

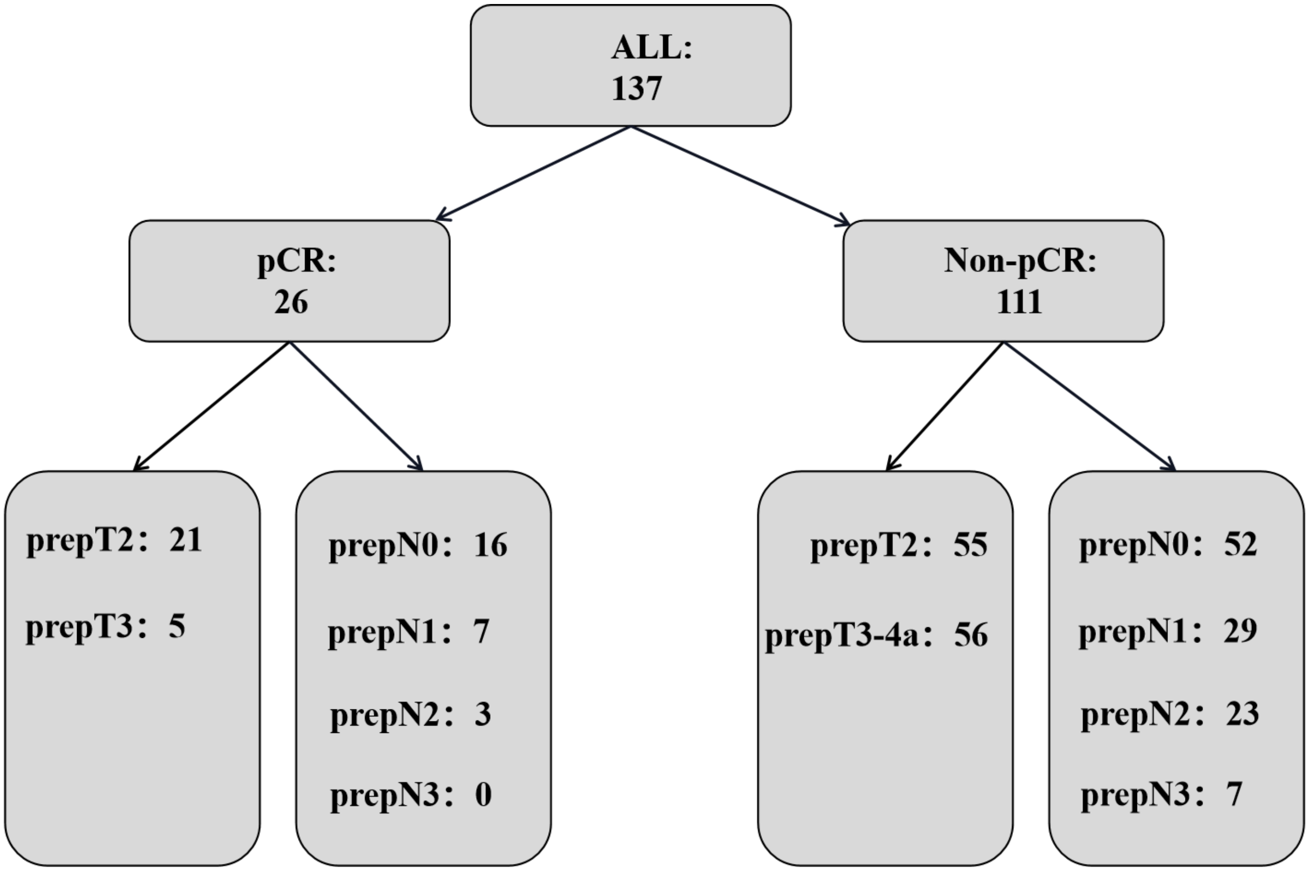

Supplementary Figure 2. The prepT and prepN stage of all 137 patients. Of 137 included patients, 26 patients had a complete pathology response (pCR) and 111 patients had a noncomplete pathology response (nonpCR). Of these 111 patients with nonpCR, 55 were prepT2 stage, and 56 were prepT3-4a stage. Of these 111 patients with nonpCR, 59 were prepN1-N3 stage.

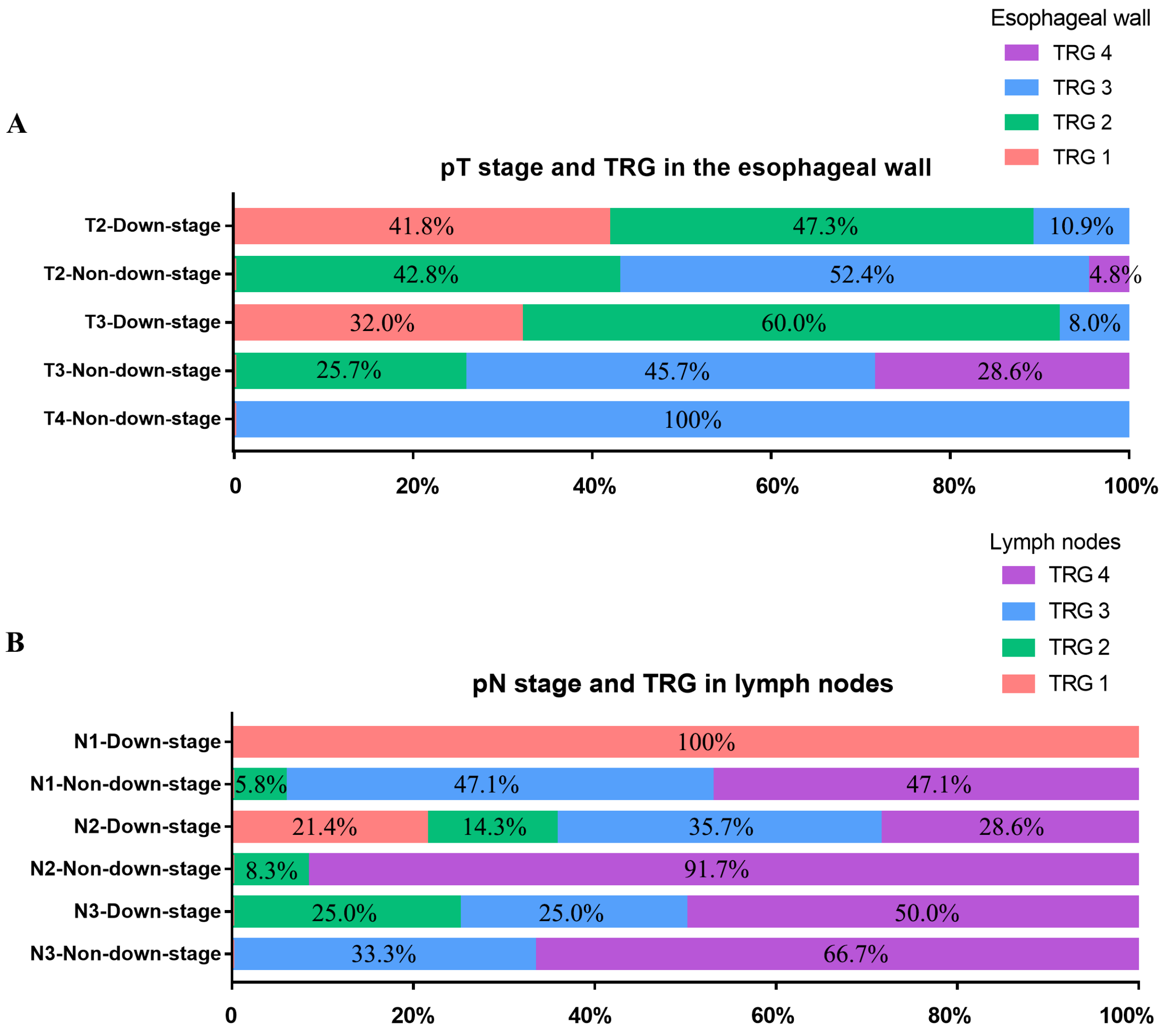

Supplementary Figure 3. A The distribution of TRG in the esophageal wall with or without ypT downstage. In the downstaged group, the percentage of TRG1 or TRG2 was higher than that in the nondownstaged group, with 89.1% vs. 42.8% in prepT2, 92% vs. 25.7% in prepT3. B The distribution of TRG in the esophageal wall with or without ypN downstage. In the downstaged group, the percentage of TRG1 or TRG2 was higher than that in the nondownstaged group, with100% vs. 5.8% in prepN1, 35.7% vs. 8.3% in prepN2, and 25.0% vs. 0 in prepN3.
