# Supplementary figures and images for: Distribution of residual tumors in esophageal squamous cell carcinoma after neoadjuvant PD-1 blockade combined with chemotherapy
Source: Front Oncol. 2023 Feb 28;13:1067897. doi: 10.3389/fonc.2023.1067897 (PMC10012861; doi:10.3389/fonc.2023.1067897)

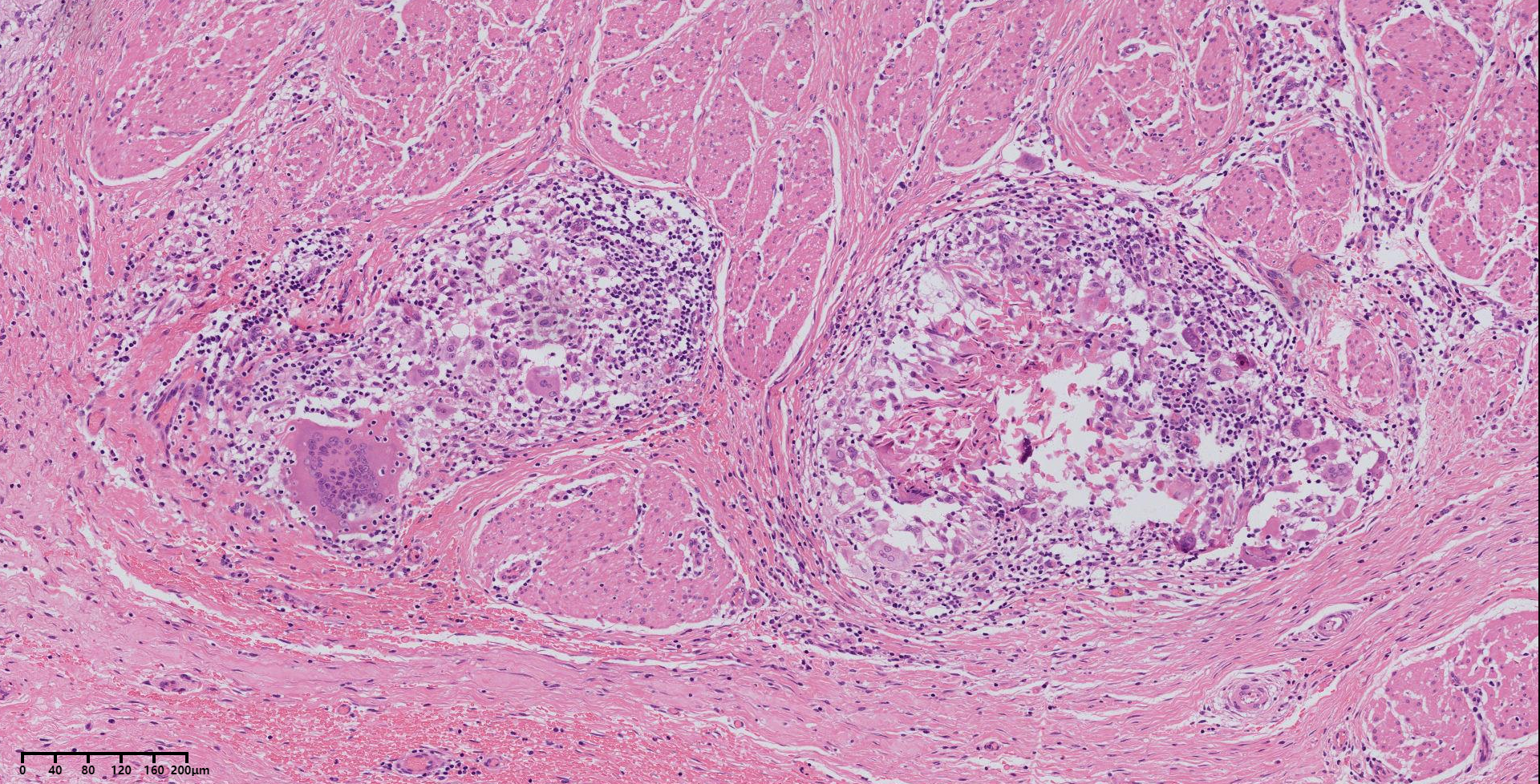

Supplement: Supplementary file 1 [file DataSheet_1.zip › Supplementary Figures/Image 1.1.TIF]

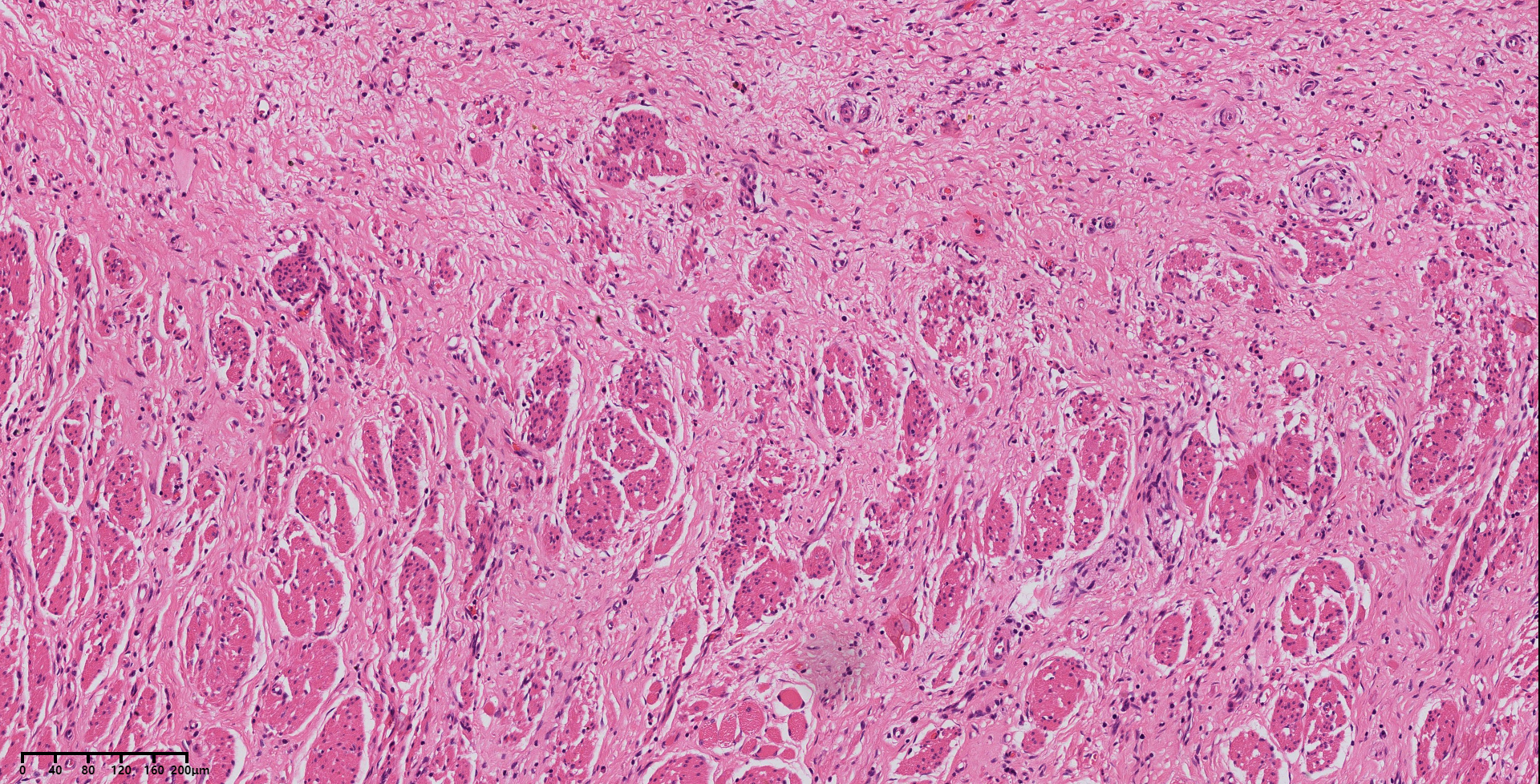

Supplement: Supplementary file 1 [file DataSheet_1.zip › Supplementary Figures/Image 1.2.TIF]

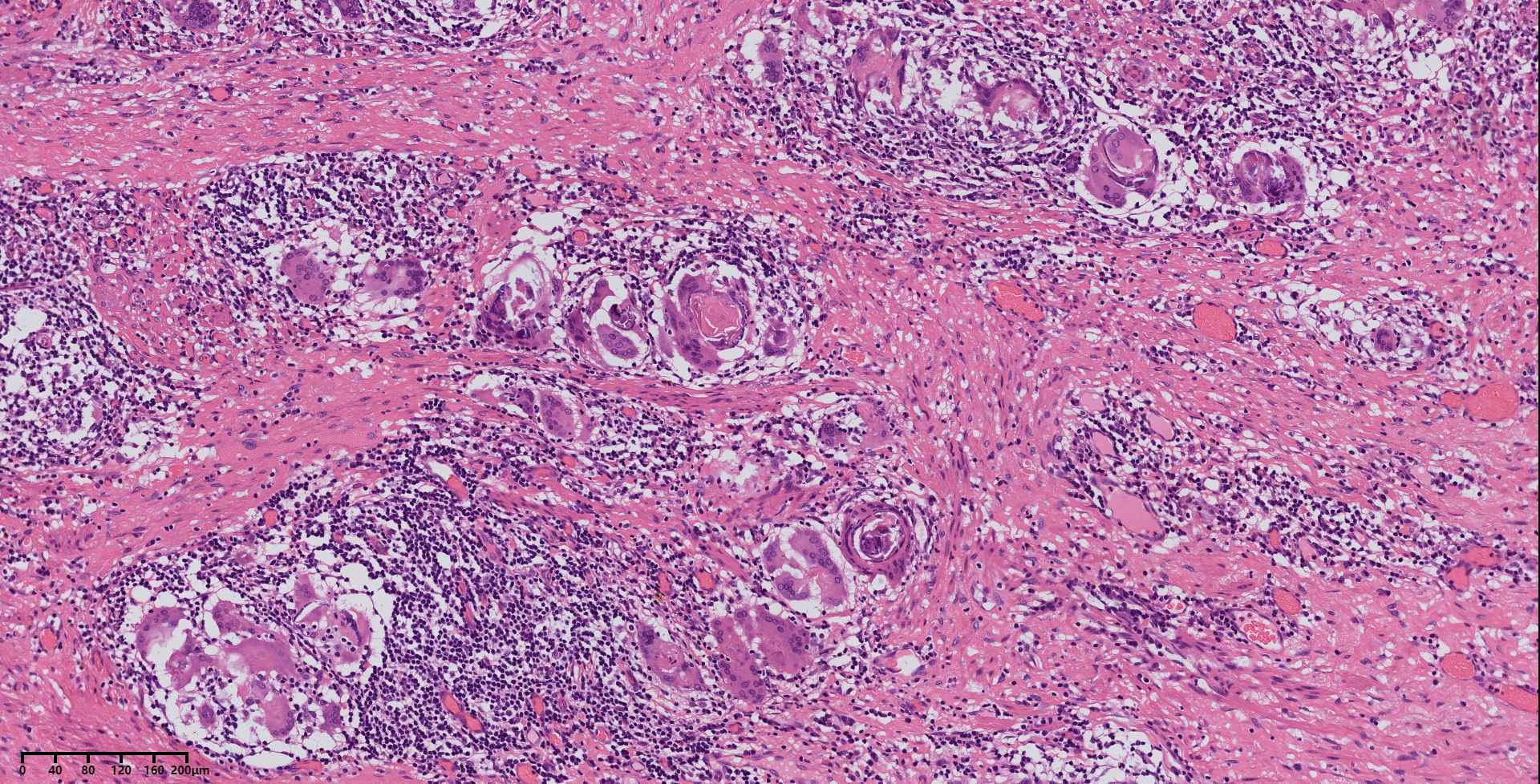

Supplement: Supplementary file 1 [file DataSheet_1.zip › Supplementary Figures/Image 1.3.TIF]

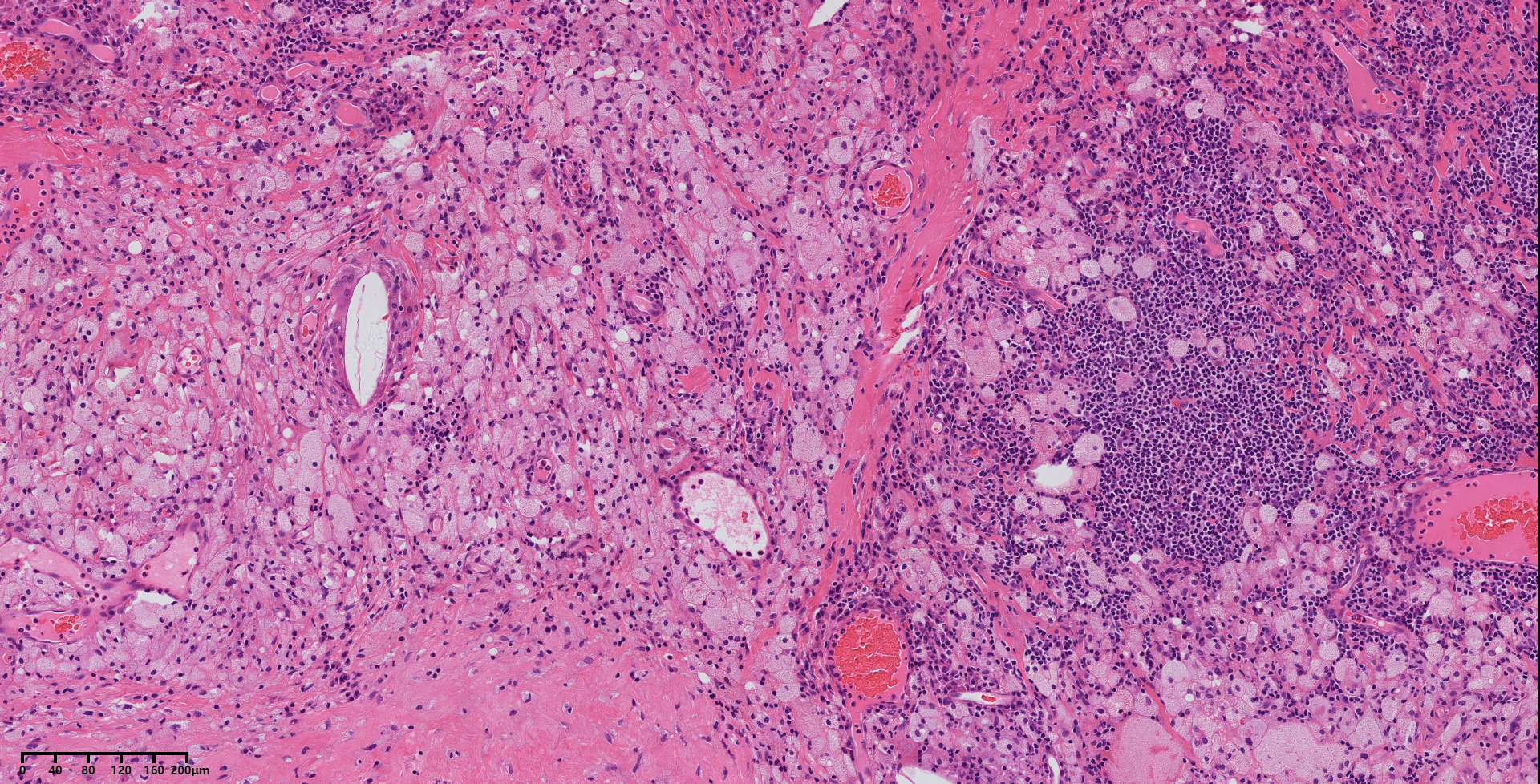

Supplement: Supplementary file 1 [file DataSheet_1.zip › Supplementary Figures/Image 1.4.TIF]

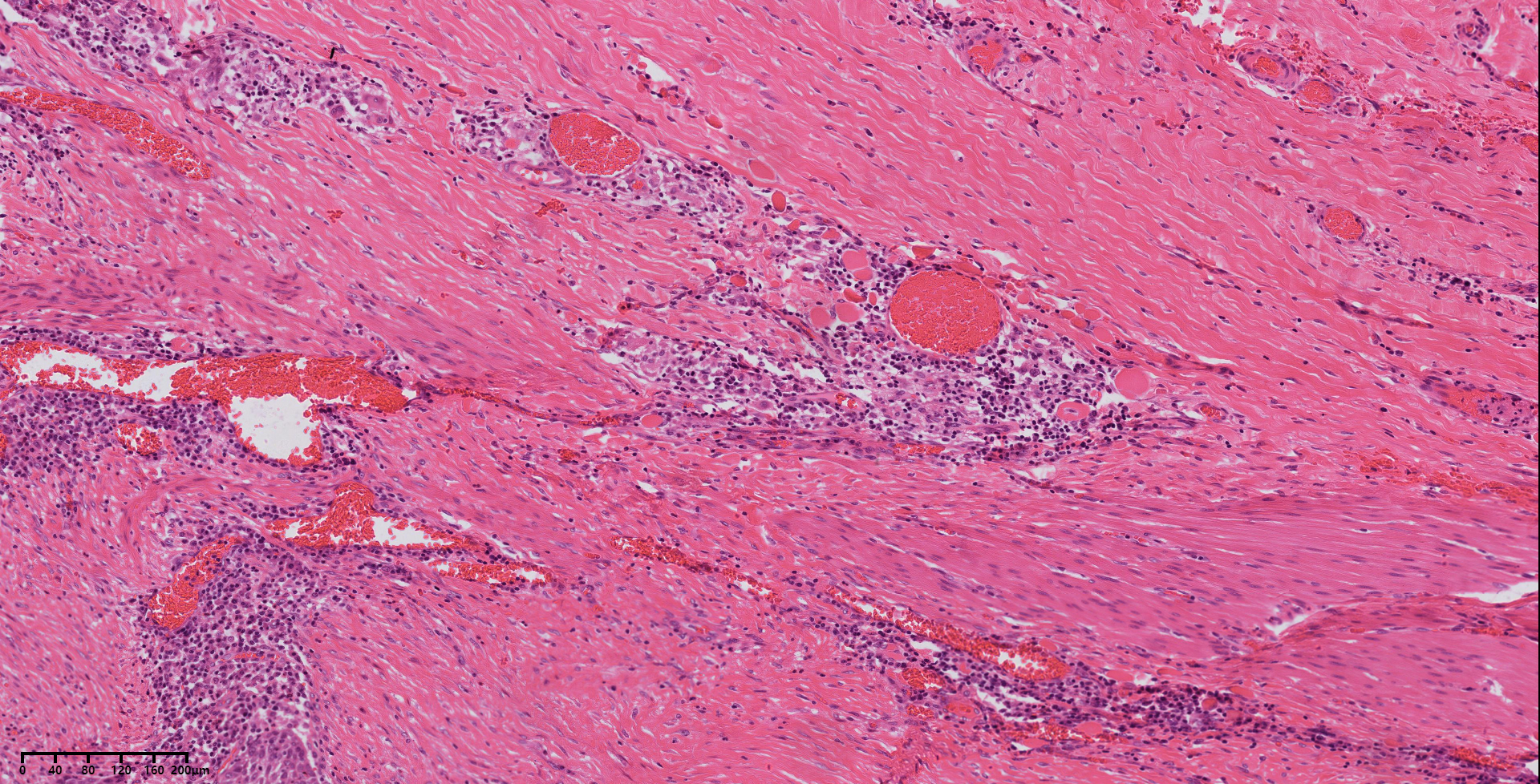

Supplement: Supplementary file 1 [file DataSheet_1.zip › Supplementary Figures/Image 1.5.TIF]

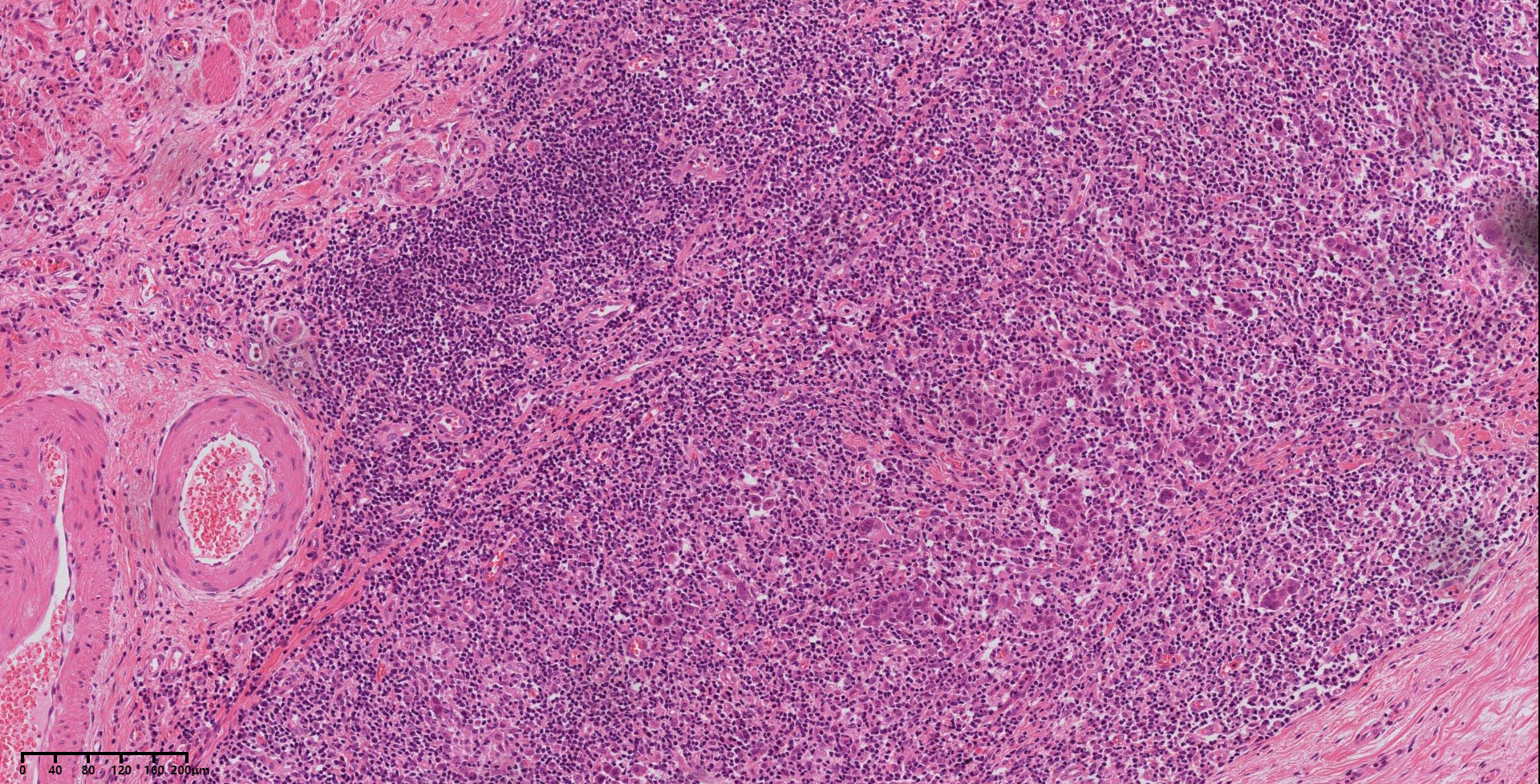

Supplement: Supplementary file 1 [file DataSheet_1.zip › Supplementary Figures/Image 1.6.TIF]

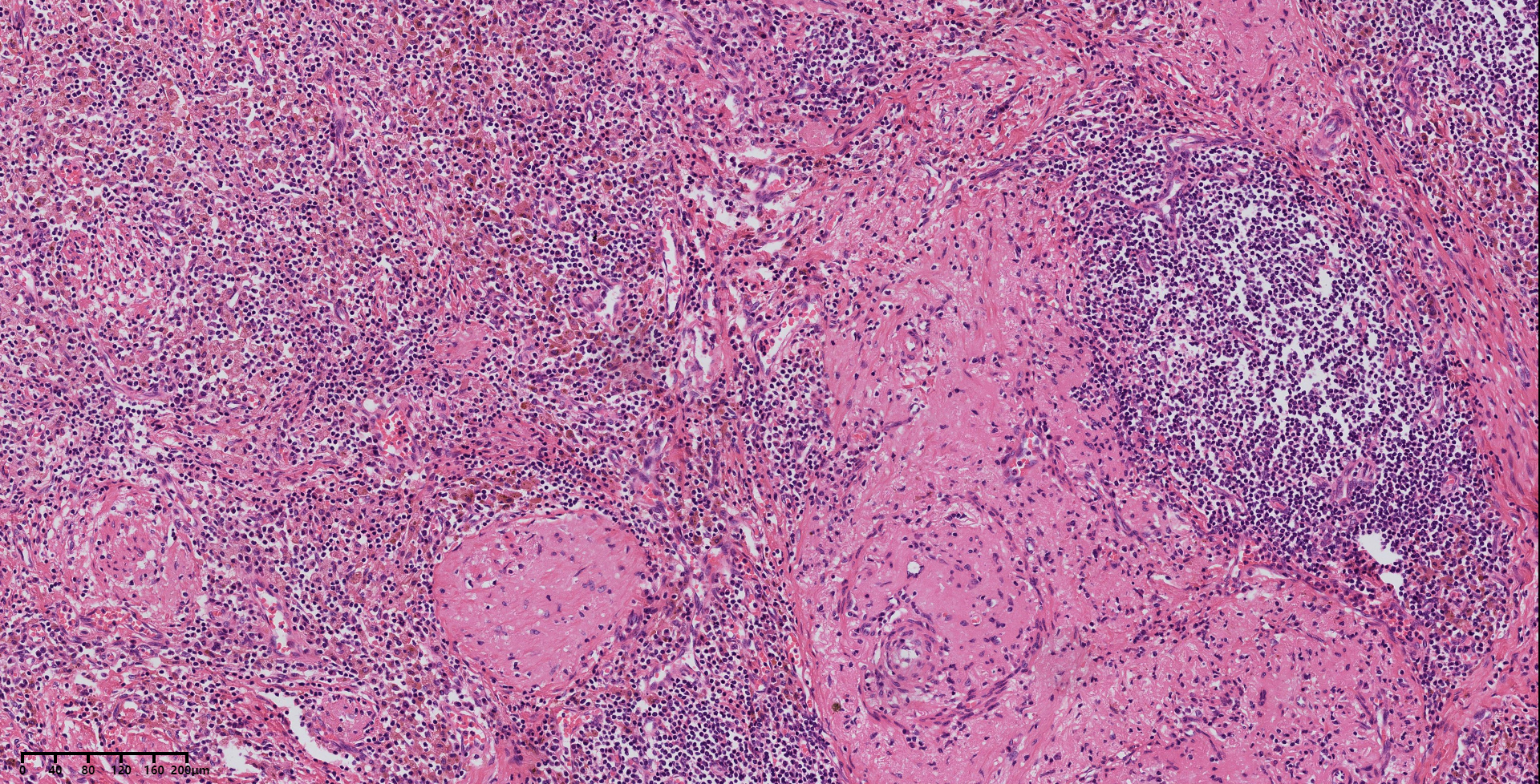

Supplement: Supplementary file 1 [file DataSheet_1.zip › Supplementary Figures/Image 1.7.TIF]

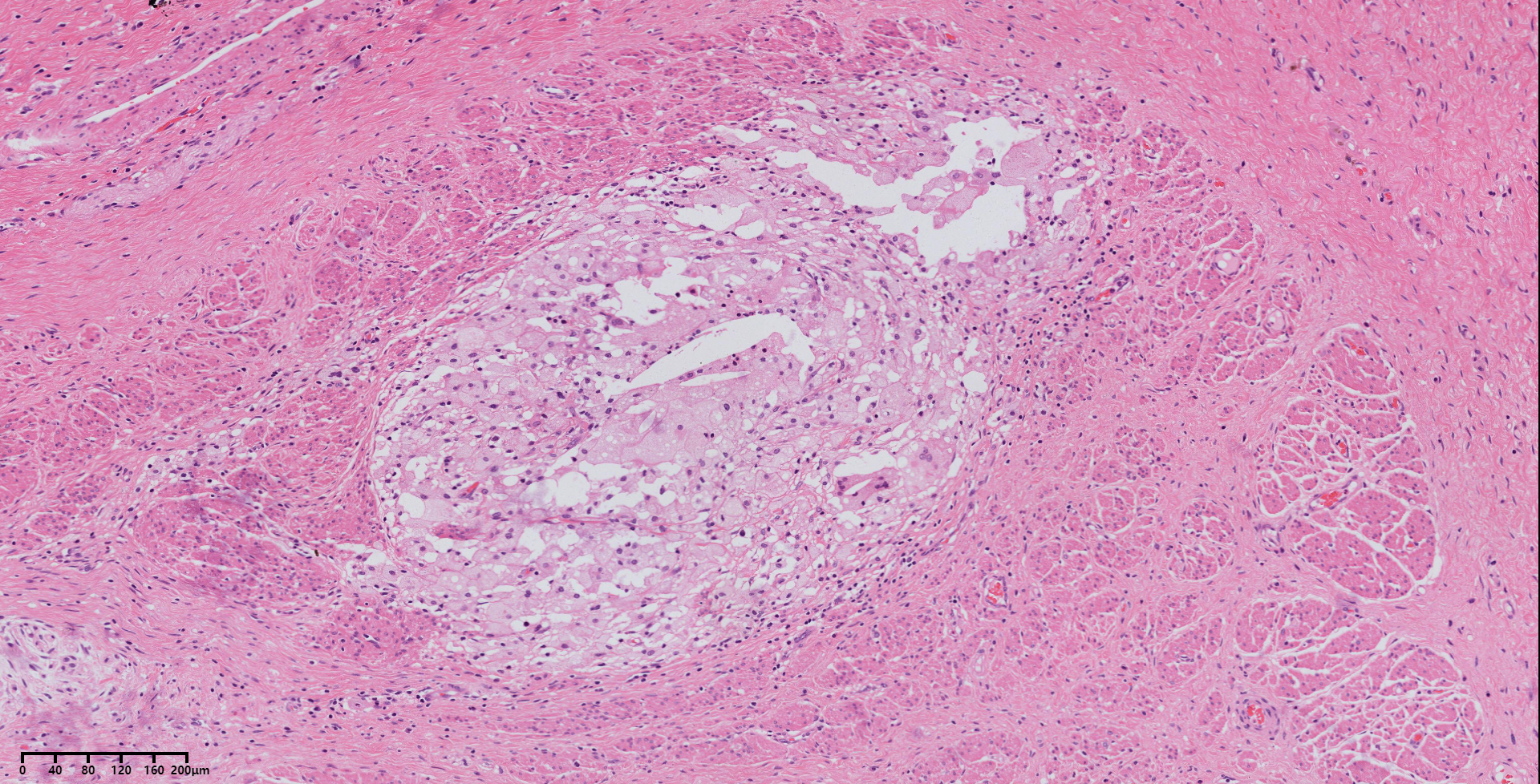

Supplement: Supplementary file 1 [file DataSheet_1.zip › Supplementary Figures/Image 1.8.TIF]
